# Perioperative Strategies in Resectable Non-Squamous Non-Small Cell Lung Cancer with EGFR Mutations and ALK Rearrangement

**DOI:** 10.3390/cancers17111844

**Published:** 2025-05-31

**Authors:** Francesco Petrella, Andrea Cara, Enrico Mario Cassina, Sara Degiovanni, Lidia Libretti, Sara Lo Torto, Emanuele Pirondini, Federico Raveglia, Francesca Spinelli, Antonio Tuoro, Stefania Rizzo

**Affiliations:** 1 Department of Thoracic Surgery, Fondazione IRCCS San Gerardo dei Tintori, 20900 Monza, Italy; francesco.petrella@irccs-sangerardo.it (F.P.); andrea.cara@irccs-sangerardo.it (A.C.); enricomario.cassina@irccs-sangerardo.it (E.M.C.); lidia.libretti@irccs-sangerardo.it (L.L.); emanuele.pirondini@irccs-sangerardo.it (E.P.); federico.raveglia@irccs-sangerardo.it (F.R.); francesca.spinelli@irccs-sangerardo.it (F.S.); antonio.tuoro@irccs-sangerardo.it (A.T.); 2 Department of Thoracic Surgery, Tor Vergata University Polyclinic, 00133 Roma, Italy; 3 Clinic of Radiology EOC, Istituto Imaging della Svizzera Italiana (IIMSI), Via Tesserete 46, 6900 Lugano, Switzerland; 4 Faculty of Biomedical Sciences, Università della Svizzera italiana (USI), Via G.Buffi 13, 6900 Lugano, Switzerland

**Keywords:** lung cancer, tyrosine kinase inhibitors (TKIs), adjuvant treatment, neoadjuvant treatment, EGFR, ALK, ROS1

## Abstract

Lung cancer is the leading cause of cancer-related death worldwide, ranking first among men and second among women for both incidence and mortality. Surgery remains the primary treatment for early-stage, resectable non-small cell lung cancer (NSCLC), encompassing stages I and selected cases of stage IIIB. For patients with stage II to III disease, as well as some stage IB tumors, neoadjuvant or adjuvant systemic therapies are recommended. It is well recognized that specific driver gene mutations play a critical role in tumor progression and aggressiveness, and patients with these genetic alterations may benefit from targeted treatment approaches. These alterations are referred to as “druggable”, “targetable”, or “actionable”, representing specific targets for personalized treatments. Tyrosine kinase inhibitors (TKIs) are now the preferred first-line treatment for patients harboring mutations in EGFR, ALK, ROS1, and BRAF. Additionally, targeted therapies exist for patients with alterations in RET, ERBB2, KRAS, MET, and NTRK, either for those who have received prior treatments or as part of ongoing clinical trials. The success of targeted therapies is reshaping treatment approaches for NSCLC with targetable driver gene alterations, both in early-stage and locally advanced settings. This review focuses on current therapeutic strategies that combine targeted therapies with surgical resection in patients with resectable non-small cell lung cancer (NSCLC) harboring actionable driver gene alterations.

## Simple Summary

Lung cancer is the leading cause of cancer-related death, ranking first in men and second in women in terms of both incidence and mortality, with an estimated 2.5 million new cases and 1.8 million deaths worldwide. Although tobacco smoking accounts for the majority of cases, about 20–25% of lung cancers develop in never-smokers, particularly in populations with low smoking rates, such as East Asian women. In these groups, elevated levels of outdoor air pollution are thought to contribute significantly to the high lung cancer incidence. The overall five-year survival rate for lung cancer remains under 20% in most countries, primarily due to difficulties in early detection. This review focuses on treatment strategies for non-small cell lung cancer (NSCLC) patients carrying specific genetic mutations.

## 1. Introduction

Lung cancer is the leading cause of cancer-related death, ranking first in men and second in women in terms of both incidence and mortality, with approximately 2.5 million new cases and over 1.8 million deaths reported worldwide [1]. Recent reports indicate that adenocarcinoma is the most common histologic type of lung cancer globally, with the highest incidence in Asia [2]. Although the majority of lung cancers are attributed to tobacco smoking, approximately 20–25% of cases are linked to other factors, particularly in regions with low smoking prevalence, such as in women from East Asia. Within this subgroup, the elevated rate of lung cancer is thought to be partially due to greater exposure to outdoor air pollution and indoor pollutants generated by the combustion of solid fuels used for cooking and heating [3,4,5]. The five-year survival rate for lung cancer is generally below 20% in most countries, largely due to the difficulty of achieving an effective early diagnosis [6,7]. Lung cancer is often diagnosed at advanced stages; specifically, about 30% of new cases are identified at a locally advanced stage, which encompasses a broad range of clinical conditions with diverse therapeutic options. Stage III non-small cell lung cancer (NSCLC) encompasses a diverse set of tumors, marked by considerable differences in tumor size, extent of local spread, and lymph node involvement [8,9]. Extensive research shows that somatic mutations in key driver genes significantly contribute to tumor progression and aggressiveness, and patients with these alterations may respond favorably to targeted treatment strategies [10].

The frequency of these genetic alterations varies across populations, affecting approximately 40–50% of NSCLC cases overall [11,12]. Activating mutations in the epidermal growth factor receptor (EGFR) gene are found in roughly 10–15% of non-small cell lung cancer (NSCLC) cases among Caucasian patients and in up to 50% of Asian individuals [13,14]. Rearrangements in the anaplastic lymphoma kinase (ALK) gene are identified in approximately 3–7% of NSCLC cases [15], while alterations involving the ROS1 gene, which encodes a receptor tyrosine kinase, are detected in around 2% of patients [16,17]. Furthermore, Kirsten rat sarcoma (KRAS) G12C mutations occur in 13% of cases [18], and MET oncogene dysregulation occurs in 5% of cases [19] Less frequently, v-Raf murine sarcoma viral oncogene homolog B (BRAF) V600 mutations (1–2%), erythroblastic oncogene B (ERBB2) mutations (2–3%), and rearranged during transfection (RET) and Neurotrophic tyrosine receptor kinase (NTRK) 1,2,3 gene rearrangements (2% and 0.3%, respectively) are reported [20,21,22,23] (Table 1).

These alterations are indicated as “druggable”, “targetable”, or “actionable”, representing specific targets for tailored therapies.

Tyrosine kinase inhibitors (TKIs) serve as the preferred first-line therapy for patients harboring mutations in EGFR, ALK, ROS1, and BRAF. Moreover, targeted treatments are either approved for previously treated individuals or are under investigation in clinical trials for genetic alterations involving RET, ERBB2, KRAS, and NTRK [24]. In contrast, non-targetable driver gene alterations are genetic mutations for which no effective targeted therapies or treatment implications are currently available [25,26]. Nevertheless, these alterations can provide valuable insights into the tumor’s biology and prognosis.

Given the significance and clinical implications of both targetable and non-targetable driver gene alterations, complete molecular profiling—such as next-generation sequencing—has become a crucial tool in the diagnostic algorithm for NSCLC. It should be performed before planning surgical resection, as part of a multimodal approach, ideally through minimally invasive methods whenever feasible [27,28]. EGFR mutations and ALK rearrangements are relatively common genetic alterations that can benefit from a combination of tyrosine kinase inhibitor (TKI) therapy and surgical resection. Thoracic surgeons are now more actively involved throughout the entire therapeutic pathway for potentially resectable tumors harboring targetable driver mutations. In these cases, the main treatment options include upfront surgery followed by adjuvant therapy, or induction therapy followed by surgical resection. These approaches require close collaboration between oncologists, surgeons, and all other professionals within the multidisciplinary team. This review aims to explore current treatment approaches that integrate targeted therapies with surgical resection in patients diagnosed with resectable NSCLC carrying actionable driver gene mutations.

## 2. EGFR

### 2.1. Adjuvant Targeted Treatments

EGFR mutations are detected in approximately 50% of NSCLC cases among Asian populations and in 10–20% of Caucasian patients [29]. Prior to the publication of the ADAURA trial results (NCT02511106) in October 2023, osimertinib was primarily used as the standard first-line treatment for EGFR-mutated advanced NSCLC, with its use in the adjuvant setting not yet established [30,31]. Osimertinib, an oral third-generation EGFR-TKI, demonstrates high selectivity and potency against EGFR-activating mutations and the resistance-associated EGFR p.Thr790Met variant, and is also effective in treating metastases within the central nervous system [32,33]. Following the results of the phase 3 FLAURA trial, osimertinib became the gold-standard therapy for previously untreated EGFR mutation-positive (Ex19del or L858R) advanced NSCLC [34,35]. Furthermore, studies show that adjuvant treatment with EGFR-TKIs in resected EGFR-positive NSCLC results in longer disease-free survival (DFS) compared to patients receiving postoperative chemotherapy or placebo [36,37].

The ADAURA trial was a phase 3, double-blind study in which resected EGFR-mutated patients (common mutations exon 21 L858R and exon 19del) were randomly assigned in a 1:1 ratio to receive either osimertinib or a placebo until disease recurrence was observed, the trial regime was completed (3 years), or a discontinuation criterion was met. In this trial, adjuvant chemotherapy was allowed, prior the randomization.

The ADAURA trial primarily evaluated investigator-assessed disease-free survival (DFS) in patients with stage II–IIIA NSCLC. The notable overall survival advantage observed is largely attributed to osimertinib’s ability to significantly lower the risk of central nervous system recurrence and to effectively target both EGFR-TKI sensitizing mutations and the T790M resistance mutation, contributing to improved tumor regression [30]. ADAURA2 is a global, phase III, randomized, double-blind, placebo-controlled trial designed to assess adjuvant osimertinib versus placebo in patients with stage IA2–IA3 EGFR-mutant NSCLC following curative-intent surgery. Participants are stratified according to pathological risk of recurrence (high vs. low), EGFR mutation subtype (exon 19 deletion vs. L858R), and racial background (Chinese Asian, non-Chinese Asian, and non-Asian). The primary outcome is disease-free survival within the high-risk group. Key secondary outcomes include DFS across the full cohort, overall survival, CNS DFS, and treatment safety. Patient enrollment began in February 2022, with interim analysis of the primary endpoint anticipated in August 2027 [38]. The ADAURA2 study extends the scope of investigation to phases IA2–IA3 and considers the pathological risk of recurrence, stratifying patients by mutation type and ethnicity.

The TARGET trial is an ongoing international, phase II, open-label, single-arm study designed to assess the long-term efficacy and safety of adjuvant osimertinib administered over five years in patients with completely resected stage II to IIIB NSCLC harboring EGFR mutations. The primary objective is to evaluate investigator-assessed disease-free survival (DFS) at five years in individuals with common EGFR mutations. Key secondary objectives include DFS at 3 and 4 years, overall survival at 3, 4, and 5 years in this group, as well as DFS across the same time points in patients with less common EGFR mutations. Additional endpoints involve safety and tolerability assessments, patterns of recurrence, and the incidence of central nervous system metastases in both mutation cohorts. Patient enrollment is ongoing, with study completion projected for 2029 [39].

The main differences from ADAURA, is the inclusion of uncommon mutations; moreover, in this trial, adjuvant chemotherapy is also allowed. Several studies conducted in recent years explored the potential role of first-generation EGFR-TKIs as adjuvant therapy in resected NSCLC. However, the results are conflicting regarding their superior effect on disease-free survival and overall survival compared to best supportive care or chemotherapy alone [9,40,41,42,43,44,45,46,47,48,49,50]. Despite progress in treatment effectiveness, metastatic relapse continues to occur frequently and is linked to poor patient prognosis, highlighting the critical need for new therapies tailored more precisely to individual molecular profiles. Importantly, the reduction in disease-free survival seen after three years in the ADAURA trial indicates that prolonging adjuvant osimertinib treatment could potentially enhance clinical outcomes. Although improving the survival of NSCLC patients is clearly a social benefit, it inevitably leads to increased direct and indirect costs—not only for genetic testing and targeted treatments, but also for the education and training of healthcare professionals [9].

Limitations of first-generation TKIs in an adjuvant setting have produced conflicting results, with no solid evidence of significant improvement compared to chemotherapy alone or best supportive care.

### 2.2. Neoadjuvant Targeted Treatments

Major pathologic response and complete pathologic response have been described as valuable surrogates for overall survival, particularly in trials evaluating neoadjuvant treatments for resectable stage I–III NSCLC patients [51]. A single-arm phase 2 trial (NCT01833572) explored the impact of neoadjuvant gefitinib—a first-generation EGFR-TKI—among EGFR-mutated, stage II to IIIA NSCLC patients [51]. The authors reported an overall response rate of 54.5% and a major pathologic response of 24.2%, with a median disease-free survival of 33.5 months [52]. A phase II trial (NCT04351555) conducted in Chinese patients with stage IIIA EGFR-mutated NSCLC compared erlotinib to cisplatin-based doublet chemotherapy. Erlotinib demonstrated superior outcomes in pathologic response rate (67% vs. 38%), overall survival (51.0 vs. 20.9 months), and overall response rate (67% vs. 19%) [53]. Likewise, the randomized, multicenter phase II EMERGING-CTONG 1103 trial evaluated erlotinib against neoadjuvant chemotherapy (gemcitabine plus cisplatin) in patients with stage IIIA N2 EGFR-mutated NSCLC. Although it did not meet its primary endpoint of overall response rate, erlotinib showed a longer median progression-free survival (21.5 vs. 11.4 months) but no significant overall survival advantage [54]. In another multicenter phase II study assessing neoadjuvant osimertinib for surgically resectable stage I–IIIA EGFR-mutated NSCLC (L858R or exon 19 deletion), 15% of patients achieved a major pathologic response, 48% showed partial response, and 44% experienced lymph node downstaging, despite the primary endpoint not being met [55]. The NEOS study, a similar single-arm, open-label phase II trial, reported an overall response rate of 71.1%, an R0 resection rate of 93.8%, and a major pathologic response rate of 10.7% [56].

Building on these promising findings and the proven benefit of combining EGFR-TKIs with chemotherapy in advanced EGFR-mutated NSCLC, further clinical trials are underway to test this combination in the neoadjuvant context [57]. Notably, the NeoADAURA trial—a phase III study—is investigating the efficacy and safety of neoadjuvant osimertinib, alone or in combination with chemotherapy, compared to chemotherapy alone in patients with resectable stage II–IIIB N2 EGFR-mutated NSCLC prior to surgery and adjuvant treatment [53]. The primary outcome is centrally assessed major pathological response at surgery, while secondary outcomes include event-free survival, pathological complete response, nodal downstaging at resection, disease-free survival, overall survival, and health-related quality of life [53].

## 3. ALK

### 3.1. Adjuvant Targeted Treatments

ALK rearrangement has been reported in about 5% of all NSCLC cases, typically in never-smokers and younger patients, diagnosed at advanced stages, and presenting a higher risk of brain metastases [58]. The efficacy of alectinib, a first-line ALK tyrosine kinase inhibitor (ALK-TKI), was established in the advanced disease setting by the phase 3 ALEX trial, which showed superior progression-free survival and overall survival compared to crizotinib, as well as better control of central nervous system metastases [59]. Following these positive outcomes, alectinib was evaluated as an adjuvant treatment post-surgery. In the phase 3, open-label, randomized ALINA trial, patients who had undergone lobectomy for non-squamous ALK-positive NSCLC were assigned to either two years of adjuvant alectinib or four cycles of platinum-based chemotherapy [60]. Those receiving alectinib experienced significantly longer disease-free survival, with a 76% reduction in the risk of recurrence or death compared to chemotherapy. Importantly, brain metastases were less frequent in the alectinib group (3.1%) versus the chemotherapy arm (11%). Although the median duration of alectinib treatment was longer (2 years compared to 2 months for chemotherapy), the rate of adverse events was comparable between the two groups. Additionally, treatment discontinuation due to adverse effects was more common with chemotherapy (12.5%) than with alectinib (5.5%) [60]. In light of the ALINA trial findings, alectinib has been approved by both the U.S. Food and Drug Administration (FDA) and the European Medicines Agency (EMA) as adjuvant therapy for patients with stage IB–IIIA ALK-positive NSCLC following curative resection.

### 3.2. Neoadjuvant Targeted Treatments

In 2019, Zhang et al. reported their initial experience with 11 patients diagnosed with ALK-positive, pathologically confirmed N2 NSCLC who received neoadjuvant crizotinib without prior therapy. Of these, ten patients achieved a partial response, while one case of grade 4 hepatic toxicity was observed. Radical surgery was successfully completed in 10 patients, with pathological complete response (pCR) documented in two cases. Disease recurrence occurred in six patients; five of them received crizotinib as first-line treatment after relapse and experienced durable responses. The authors concluded that preoperative crizotinib is both feasible and well tolerated in ALK-positive, locally advanced NSCLC patients eligible for surgical resection. Moreover, neoadjuvant crizotinib effectively eradicated circulating molecular residual disease without compromising the effectiveness of subsequent first-line crizotinib therapy [61]. A retrospective analysis comparing stage III ALK-positive NSCLC patients undergoing radical resection after neoadjuvant therapy with either alectinib (16 patients) or crizotinib (13 patients) demonstrated higher pathological complete response rates in the alectinib group (37.5% vs. 15.4%) [62]. Early findings from the NAUTIKA 1 trial reported a 100% complete resection rate following standard lung surgery in ALK-positive NSCLC patients treated with neoadjuvant alectinib [63]. The ongoing ALNEO trial is enrolling patients with potentially resectable stage III ALK-positive NSCLC (any T with N2, or T4N0-1) to receive neoadjuvant oral alectinib followed by surgery and adjuvant alectinib for 24 cycles (96 weeks). Its primary endpoint is major pathological response, with secondary endpoints including pathological complete response, objective response rate, event-free survival, disease-free survival, overall survival, and safety [64] (Table 2).

## 4. The Role of Immunotherapy in Resectable Lung Cancer

Nowadays, surgery remains the cornerstone treatment for early-stage, resectable NSCLC. For patients with stage II to III disease, neoadjuvant or adjuvant systemic therapies are routinely employed. Tyrosine kinase inhibitors (TKIs) are recommended for individuals harboring targetable driver mutations. However, a comprehensive management strategy should also consider PD-L1 expression levels, available immunotherapy options, and the potential role of radiotherapy. In the adjuvant setting, maintenance therapy with immune checkpoint inhibitors—such as atezolizumab or pembrolizumab—is advised for one year following chemotherapy, with treatment decisions guided by both PD-L1 status and molecular profiling. Meanwhile, neoadjuvant treatment has evolved to incorporate concurrent chemo-immunotherapy regimens as the standard of care, sometimes followed by adjuvant immunotherapy.

This therapeutic approach may also benefit patients with actionable mutations, including those involving EGFR or ALK. It is now well documented that patients diagnosed with NSCLC who have targetable driver gene alterations do not gain maximal benefit from immunotherapy, with the exception of KRAS mutations. Similarly, combining immunotherapy with targeted therapy does not provide an additional advantage [65,66,67].

It is important to highlight the risk of neoadjuvant treatment failure, which could lead to surgery cancellation in non-responding patients. Therefore, induction treatments, which are well established in locally advanced stage III patients, should be carefully tailored for earlier stages, such as stage II. Moreover, toxicity must be closely monitored when defining the therapeutic strategy, particularly in the curative setting. For example, crizotinib (an ALK TKI) has been associated with an increased risk of liver toxicity after chemo-immunotherapy, and sotorasib (a KRAS inhibitor) can also pose risks when used shortly after immune checkpoint inhibitors. Similarly, osimertinib has shown an increased risk of pneumonitis when administered after chemo-immunotherapy [65,66,67]. Building on advances made in the metastatic setting, newer TKIs targeting EGFR, ALK, RET, BRAF, ROS1, NTRK, KRAS, and MET have demonstrated enhanced penetration of the central nervous system. Osimertinib and alectinib, in particular, retain this ability even when administered as adjuvant therapy. Patients treated with targeted agents alongside surgery require rigorous postoperative monitoring, with computed tomography remaining the preferred imaging modality for NSCLC surveillance. Alternative imaging methods may be utilized depending on specific histologic and clinical characteristics [65,66,67]. Multiple randomized clinical trials have confirmed the survival advantage of EGFR-TKIs used as neoadjuvant treatment. Such induction therapies can shrink tumor size, improve the chances of complete surgical resection, and reduce the likelihood of disease recurrence.

However, it also carries potential drawbacks, such as delaying surgery and allowing for possible disease progression during the treatment period. Additionally, both chemo-immunotherapy and TKI-based induction therapies can significantly increase surgical complexity. They may obscure anatomical planes, raise the conversion rate from minimally invasive to open surgery, and, in some cases, make a minimally invasive approach inadvisable from the outset [67].

Currently, the objective response rate and safety profile of TKIs as induction therapy for stage II–IIIA NSCLC are well established. TKIs can induce significant tumor shrinkage and improve the rate of radical resection. However, definitive evidence regarding their impact on overall survival and recurrence risk remains lacking. Further large-scale randomized controlled trials are required to better define the role of TKIs in both neoadjuvant and adjuvant treatment settings [67].

## 5. Role of Imaging

Computed tomography (CT) imaging is widely used across various clinical settings [68,69,70,71,72], and it plays specifically a central role in the diagnostic workup and monitoring of treatment response in lung cancer patients [70,71,72], including those bearing targetable mutations. Pre-treatment CT scans may provide detailed information on tumor size, location, presence of metastatic disease, in the lymph nodes as well as in distant organs, thus allowing for assessment of baseline extent of the disease. During treatment, serial imaging helps monitoring treatment response to EGFR-TKIs. In this context, thanking the central nervous system penetration of osimertinib, CT scans (supplemented by brain magnetic resonance imaging in case of doubtful findings) can be particularly useful in detecting brain metastases, a common site of recurrence in EGFR-mutant NSCLC. In clinical practice, CT imaging can track tumor shrinkage and helps evaluate potential resistance mechanisms.

Applications of artificial intelligence aiming at predicting the presence of targetable mutations show promising results. Among these, the vast majority of authors have assessed associations between specific CT features and gene mutations [72], often relying on radiomics/radiogenomics, and using machine learning techniques [73,74,75]. While many of these studies demonstrate the potential to predict the presence of mutations based on radiomics features extracted through dedicated software [76], challenges related to the quality, reproducibility, and generalizability of the results are still present. Therefore, further research is warranted to address these issues before these methods can be integrated into the clinical practice [77,78,79,80].

## 6. Conclusions

In conclusion, worldwide progress in targeted therapies is reshaping the management of NSCLC with actionable driver mutations across early and locally advanced stages. The broad adoption of comprehensive preoperative genomic profiling, including in early-stage disease, deepens insights into tumor biology and improves the precision of predicting therapeutic responses.

It is expected that targeted treatments—together with immunotherapy—will play a crucial role in the future, even in surgical settings.

## Figures and Tables

**Table 1:** The most common NSCLC targetable driver gene alterations and their frequency.

Targetable Driver Gene Alterations	Frequency
*EGFR*	10–15% ^(1)^ 50% ^(2)^
*ALK*	3–7%
*ROS1*	2%
*KRAS G12C*	13%
*MET*	5%
*BRAFV600*	1–2%
*ERBB2*	2–3%
*RET*	2%
*NTRK1,2,3*	0.3%

^(1)^ Caucasian ^(2)^ Asian.

**Table 2:** Ongoing clinical trials with targeted therapies in the preoperative setting.

Trial	Phase	Stage	Mutation	Treatment	Duration of Neoadjuvant Treatment	Primary End Point
NCT04351555 (NeoADAURA)	III	II–IIIB N2	EGFR	osimertinib + platinum-based chemotherapy vs. placebo + platinum-based chemotherapy or osimertinib monotherapy	3 cycles	Major pathological response
NCT03203590	III	II–IIIA	EGFR	platinum-based chemotherapy vs. gefitinib	2 cycles 8 weeks	2-year disease-free survival
NCT05011487 (NOCE01)	II	IIIA T3-4N2 IIIB	EGFR	osimertinib + platinum-based chemotherapy	60 days (2 cycles)	Complete lymph node clearance rate (ypN0)
NCT06268210	II	IB–IIIB	EGFR	lazertinib or lazertinib, pemetrexed, carboplatin	3 years 3 cycles	Primary pathological response
NCT05469022 (NeolazBAL)	II	I–IIIB IVA (single metastasis)	EGFR	lazertinib	9 weeks	Objective response rate at 9 weeks
NCT05104788	II	IIA–IIIB	EGFR	icotinib + platinum-based chemotherapy	2 cycles	Major pathological response
NCT03749213	II	IIIA (N2)	EGFR	icotinib	8 weeks	Objective response rate
NCT05132985	II	II–IIIB (N2)	EGFR	icotinib + platinum-based chemotherapy	2 cycles	Major pathological response
NCT05987826	II	II–IIIB (T3N2)	EGFR	furmonertinib	8 weeks	Objective response rate at 8 weeks
NCT05430802 (FORESEE)	II	IIIA/IIIB	EGFR	furmonertinib + platinum-based chemotherapy	3 cycles	Objective response rate
NCT04685070	II	III	EGFR	almonertinib	2–4 cycles (4 weeks per cycle)	Objective response rate
NCT04455594 (ANSWER)	II	IIIA (N2)	EGFR	almonertinib Investigator-choice therapy (erlotinib or chemotherapy)	3 cycles	Objective response rate
NCT05015010 (ALNEO)	II	III (any T,N2, T4N0-1)	ALK	alectinib	8 weeks	Major pathological response
NCT04302025 (NAUTIKA-1)	II	IB, IIA, IIB, IIIA IIIB(T3N2)	ALK ROS1 NTRK1/2/3 BRAF RET	alectinib entrectinib pralsetinib divarasib	8 weeks	Major pathological response
NCT06282536 (LungMate-018)	II	III–IVA	ALK	iruplinalkib	4 cycles	Objective response rate
NCT05380024 (NEOEAST)	II	IIA–IIIB	ALK	ensartinib	8 weeks	Major pathological response
NCT05472623 (Neo-KAN)	II	IB–IIIA	KRAS	adagrasib or adagrasib/ nivolumab	6 weeks	Pathological complete response
NCT05118854	II	IIA–IIIB (T3-4N2)	KRAS	sotorasib + platinum-based chemotherapy	4 cycles	Major pathological response
NCT06054191	II	IB–IIIA IIIB (T3N2, T4N2)	BRAF MET	dabrafenib and trametinib (BRAF cohort) capmatinib (MET cohort)	8 weeks	Pathological complete response

Modified from: Fuorivia et al. [9].
